# Clinical utility of quantitative analysis of bone scintigraphy in detecting clinically active joint and high disease activity in patients with rheumatoid arthritis

**DOI:** 10.1186/s12880-021-00712-2

**Published:** 2021-11-23

**Authors:** Jeong Won Lee, Sung Hae Chang, Su Jin Jang, Hee Jin Park, Sang Mi Lee, Ki Jin Jung

**Affiliations:** 1 Department of Nuclear Medicine, Catholic Kwandong University College of Medicine, International St. Mary’s Hospital, 25 Simgok-ro 100-gil, Seo-gu, Incheon, 22711 Korea; 2 Division of Rheumatology, Department of Internal Medicine, Soonchunhyang University Cheonan Hospital, 31 Suncheonhyang 6-gil, Dongnam-gu, Cheonan, 31151 Chuncheongnam-do Korea; 3 Department of Nuclear Medicine, CHA Bundang Medical Center, CHA University, 59 Yatap-ro, Bundang-gu, Seongnam-si, 13496 Gyeonggi-do Korea; 4 Division of Rheumatology, Department of Internal Medicine, Catholic Kwandong University College of Medicine, International St. Mary’s Hospital, 25 Simgok-ro 100-gil, Seo-gu, Incheon, 22711 Korea; 5 Department of Nuclear Medicine, Soonchunhyang University Cheonan Hospital, 31 Suncheonhyang 6-gil, Dongnam-gu, Cheonan, 31151 Chungcheongnam-do Korea; 6 Department of Orthopedic Surgery, Soonchunhyang University Cheonan Hospital, 31 Suncheonhyang 6-gil, Dongnam-gu, Cheonan, 31151 Chungcheongnam-do Korea

**Keywords:** Bone scintigraphy, Rheumatoid arthritis, Joint, Quantitative analysis, DAS28

## Abstract

**Background:**

The purpose of this study was to investigate the efficiency of quantitative parameters of bone scintigraphy in detecting clinically active joint and high disease activity in patients with rheumatoid arthritis.

**Methods:**

We retrospectively enrolled 65 patients with rheumatoid arthritis who underwent bone scintigraphy for diagnostic work-up. Quantitative analysis of bone scintigraphy images was conducted using an in-house software, and joint uptake ratio of 28 joints was measured for the calculation of the disease activity score of 28 joints using erythrocyte sedimentation rate (DAS28-ESR). The relationship between joint uptake ratio and clinical findings and the efficiency of joint uptake ratio in detecting clinically active joint and high disease activity were assessed.

**Results:**

Clinically active joint (tender and/or swollen joints) showed significantly higher joint uptake ratio than did other non-affected joints (*p* < 0.05). The sensitivity, specificity, positive predictive value, and negative predictive value (NPV) of joint uptake ratio for identifying clinically active joint were 78.7%, 52.0%, 32.9%, and 89.1%, respectively, and those of the summed joint uptake ratio for detecting high disease activity were 92.9%, 66.8%, 43.3%, and 97.1%, respectively; the joint uptake ratio showed high detection ability, especially for active joints of the elbow, wrist, and metacarpo-phalangeal joint areas. The summed joint uptake ratio of 28 joints showed a significantly strong positive correlation with DAS28-ESR (*p* < 0.001; correlation coefficient, 0.725).

**Conclusion:**

Quantitative parameters of bone scintigraphy showed high sensitivity and NPV for detecting clinically active joint and high disease activity in patients with rheumatoid arthritis.

## Background

Rheumatoid arthritis is a chronic systemic autoimmune inflammatory disease with a global prevalence of 1% [1, 2]. Rheumatoid arthritis is characterized by chronic synovial membrane inflammation, which progressively leads to joint destruction, bone erosion, and functional disability [1, 3]. The treat-to-target concept has been considered as the principle of treatment strategy for rheumatoid arthritis; it aims to achieve early clinical remission or low disease activity with early intervention and to sustain it by regular measurement of disease activity [2, 4]. To provide prompt and appropriate treatment to patients with rheumatoid arthritis, it is important to accurately estimate the disease activity [4, 5]. Currently, diverse kinds of tools such as disease activity score of 28 joints (DAS28), health assessment questionnaire, and clinical disease activity index have been used to measure the disease activity of rheumatoid arthritis [4, 5]. However, the scores obtained using these tools are mainly dependent on the patient’s subjective symptoms, and only 58% of physicians reported that they used quantitative measurement of the disease activity during most visits mainly due to the lack of time [5, 6]. Therefore, in clinical practice, various imaging modalities that can evaluate the presence and degree of joint inflammation have been used to detect synovitis and to estimate the disease activity in patients with rheumatoid arthritis [6, 7].

Bone scintigraphy using Tc-99 m labeled diphosphonate has been widely used for the diagnosis of various bone and joint diseases owing to its high sensitivity in detecting bone and joint pathology, good availability, and ability to provide whole-body images with a single scan, [8–11]. Furthermore, because increased radiotracer uptake is observed in an inflamed joint, previous studies have demonstrated that bone scintigraphy could help identify joints with synovitis and diagnose rheumatoid arthritis [6, 12]. However, until the present day, visual assessment has been primarily used for the interpretation bone scintigraphy images of patients with joint symptoms, which intrinsically involves significant inter-reader discrepancies [6, 10, 12]. Although quantitative analytical methods have been introduced for specific joint areas such as the sacroiliac and temporomandibular joints, only a few studies have evaluated the clinical value of quantitative analysis of whole-body joint areas on bone scintigraphy images in patients with arthritis diseases [10, 13–16]. In this regard, we have developed an in-house software that can automatically measure the uptake of 64 joints in 14 joint areas (shoulder, sternoclavicular, elbow, wrist, metacarpo-phalangeal [MCP], thumb interphalangeal [IP], hand proximal and distal IP, sacroiliac, knee, ankle, tarsal, metatarso-phalangeal, and foot IP joints) in our previous study and demonstrated a substantial inter-rater agreement for the quantitative parameter of bone scintigraphy; inter-rater reliability of this software-based assessment was higher than that of visual assessment [10].

In the present study, using our in-house software, we performed quantitative analysis of bone scintigraphy images acquired from patients with rheumatoid arthritis and investigated whether the quantitative parameters of bone scintigraphy could have a clinical value in identifying clinically active joint and detecting high disease activity in patients with rheumatoid arthritis.

## Methods

### Patients

**Fig. 1:**
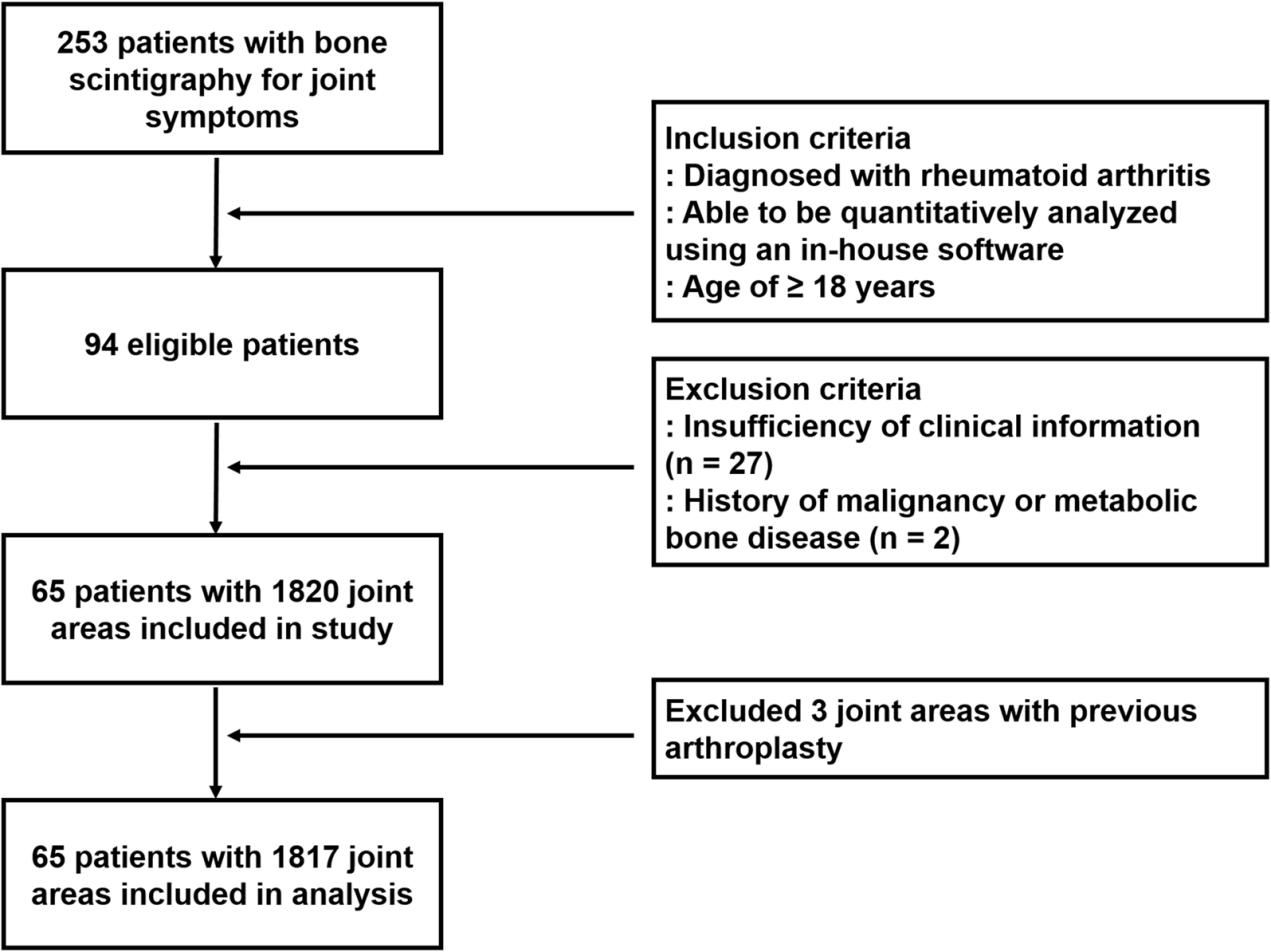
Inclusion and exclusion of patients in the study

We retrospectively reviewed the electronic medical records of 253 patients who underwent bone scintigraphy for evaluating their joint symptoms (pain, tenderness, and/or swelling) in Soonchunhyang University Cheonan Hospital between January 2019 and November 2020. Among them, 65 patients were included in the present study according to the following inclusion criteria (Fig. 1): (1) diagnosis of rheumatoid arthritis according to the 2010 American College of Rheumatology/European League Against Rheumatism classification criteria for rheumatoid arthritis, (2) presence of bone scintigraphy images which were able to be quantitatively analyzed using our in-house software, specifically, those containing whole-body anterior and spot images that properly included 14 joint areas (shoulder, sternoclavicular, elbow, wrist, MCP, thumb IP, hand proximal and distal IP, sacroiliac, knee, ankle, tarsal, metatarso-phalangeal, and foot IP joints) [10], and (3) patient age of ≥ 18 years. The following were the exclusion criteria: (1) insufficiency of information for calculating DAS28 using erythrocyte sedimentation rate (DAS28-ESR) and (2) a previous medical history of malignancy or metabolic bone disease. For the diagnostic work-up of the patients, physical examination and laboratory biochemical tests, including measurement of serum ESR, rheumatoid factor, and anti-citrullinated peptide antibody (ACPA), as well as bone scintigraphy were performed. On clinical examination, joints that showed tenderness and/or swelling were defined as clinically active joints. Based on the results of these diagnostic examinations, the disease activity of rheumatoid arthritis at the time of initial diagnosis was determined by calculating the DAS28-ESR. According to the DAS28-ESR values, all patients were classified into three groups: patients with low disease activity (DAS28-ESR ≤ 3.2), moderate disease activity (3.2 < DAS28-ESR ≤ 5.1), or high disease activity (DAS28-ESR > 5.1).

For comparison, 15 patients with osteoarthritis (12 men and 3 women; median age, 57 years [range, 42–78 years]) were selected from the 253 patients with initial screening and were also included in the study as a comparison patient group.

The study was and approved by the Institutional Review Board of our medical center, and the study was conducted according to the guidelines of the Declaration of Helsinki as revised in 2013 and its later amendments. Patient consent was waived by the Institutional Review Board due to the retrospective nature of the study.

### Bone scintigraphy and quantitative analysis

A dual-head gamma camera (Infinia GP, GE Healthcare, Milwaukee, WI, USA) with a low-energy general-purpose collimator was used for bone scintigraphy imaging. In all patients, Tc-99 m methylene diphosphonate of 740–925 MBq was intravenously administered. Approximately 3 h after the injection, anterior and posterior whole-body images were acquired using the continuous acquisition mode at a scanning speed of 12 cm/min, and subsequently, spot images of the posterior pelvic area, bilateral hands, and bilateral feet were acquired [10].

Bone scintigraphy images of all enrolled patients were quantitatively analyzed using an in-house software. As described in our previous study [10], our in-house software automatically measured the mean values of joint uptake in the 14 joint areas and the reference bone uptake values on whole-body anterior images and spot images of the posterior pelvic area, bilateral hand, and bilateral feet. Afterwards, with mean joint uptake and the reference bone uptake, the software automatically calculated the joint-to-bone uptake ratio (joint uptake ratio) for each joint [10]. Among the joints in the 14 joint areas, joint uptake ratios of 28 joints that were used for calculating DAS28 (bilateral shoulder, elbow, wrist, and knee joints, 10 MCP joints, bilateral thumb IP joints, and 8 hand proximal IP joints) were recorded and subjected to statistical analysis. For the 65 patients with rheumatoid arthritis, joint uptake ratio of 1817 joints were finally included in the statistical analysis, because two knee joint areas and one elbow joint area were excluded from the quantitative analysis owing to previous arthroplasty (Fig. 1). For the 15 patients with osteoarthritis in the comparison group, joint uptake ratio of 420 joints were included in the analysis. Furthermore, for each patient, the summed joint uptake ratio of the 28 joints was calculated.

### Statistical analysis

To evaluate the differences in the joint uptake ratio and summed joint uptake ratio between groups, analysis of variance with post-hoc comparisons using Scheffe test and the Kruskal–Wallis test with post-hoc comparisons with Dunn’s test were performed according to the results of Levene’s test. For continuous variables included in the correlation analysis, Shapiro–Wilk test was performed to evaluate the normality of distribution, and Pearson’s correlation coefficients were calculated to investigate the relationship of the joint uptake ratio with clinical factors. The detection ability of the joint uptake ratio for clinically active joint and that of the summed joint uptake ratio of 28 joints for high disease activity were evaluated based on the area under the receiver operating characteristic (ROC) curve (AUC) values. A bootstrap method was used to estimate the 95% confidence interval (CI) for the AUC values. The specific cut-off values of the joint uptake ratio and summed joint uptake ratio of 28 joints were identified using the Youden index. Using the specific cut-off values, the sensitivity, specificity, positive predictive value, and negative predictive value of the joint uptake ratio for identifying clinically active joint and those of summed joint uptake ratio for detecting high disease activity were assessed. MedCalc Statistical Software (version 20, MedCalc Software Ltd, Ostend, Belgium) was used for the statistical analysis. P-values of < 0.05 was regarded as statistically significant.

## Results

### Clinical features of patients

The clinical characteristics of the study participants are shown in Table 1. All enrolled patients underwent bone scintigraphy for the initial work-up of rheumatoid arthritis. Among the 65 patients, 48 (73.8%) and 28 (43.1%) patients were positive for rheumatoid factor and ACPA, respectively. Physical examination revealed that, among the 1817 joints included for calculating DAS28-ESR, there were 174 tender joints (9.6%), 44 swollen joints (2.4%), and 200 joints that were both tender and swollen (11.0%); thereby, 418 joints (23.0%) were clinically active joints. Based on the assessment of baseline disease activity with DAS28-ESR at the time of diagnosis of rheumatoid arthritis, patients were classified as follows: high disease activity (DAS28-ESR > 5.1), 14 patients (21.5%); moderate disease activity (3.2 < DAS28-ESR ≤ 5.1), 34 patients (52.3%); and low disease activity (DAS28-ESR ≤ 3.2), 17 patients (26.2%).

**Table 1:** Baseline characteristics of the patients (n = 65)

Characteristics	Median value (range)
Age (years)	
Sex	56 (19–83 years)
Men	18 (27.7%)*
Women	47 (72.3%)*
Disease activity	
Number of tender joints among 28 joints for each patient	4 (1–20)
Number of swollen joints among 28 joints for each patient	2 (1–14)
Serum ESR level (mm/h)	14 (2–120)
Patient global assessment	45 (20–90)
DAS28-ESR	4.02 (1.61–6.90)
Bone scintigraphy	
Joint uptake ratio	1.14 (0.10–7.46)
Summed joint uptake ratio of 28 joints	35.70 (18.19–50.53)

DAS28-ESR, disease activity score in 28 joints using erythrocyte sedimentation rate; ESR, erythrocyte sedimentation rate

^*^Number of patients (%)

### Joint uptake ratio for detecting clinically active joint

**Fig. 2:**
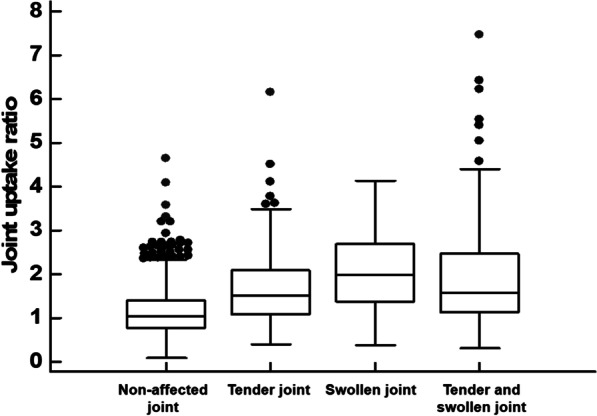
Distribution of the joint uptake ratio in non-affected joints (1399 joints), tender joints (174 joints), swollen joints (44 joints), and both tender and swollen joints (200 joints)

Joint uptake ratios on bone scintigraphy were compared among 174 tender joints, 44 swollen joints, 200 both tender and swollen joints, and 1399 non-affected joints (Fig. 2). Kruskal–Wallis test demonstrated that there were significant differences in joint uptake ratios among the four joint groups (*p* < 0.001). Post-hoc comparison showed that tender joints (1.68 ± 0.81), swollen joints (2.07 ± 0.92), and both tender and swollen joints (1.94 ± 1.16) showed significantly higher values of joint uptake ratio than did non-affected joints (1.12 ± 0.49; *p* < 0.05 for all). Meanwhile, there were no significant differences in joint uptake ratios among tender joints, swollen joints, and both tender and swollen joints (*p* > 0.05 for all).

Joint uptake ratios in the enrolled 65 patients were also compared with joint uptake ratios in the 15 patients of the comparison group with osteoarthritis. Among 420 joints of the comparison patient group, there were 26 affected joints (joint uptake ratio of 1.94 ± 0.90) and 394 non-affected joints (joint uptake ratio of 0.88 ± 0.35). In comparisons with affected joints of the comparison patient group, tender joints, swollen joints, and both tender and swollen joints showed no significant differences of joint uptake ratio (*p* > 0.05 for all), and joints without tenderness and swelling showed significantly lower values of joint uptake ratio (*p* < 0.05). In comparisons with non-affected joints of the comparison group, joints without tenderness and swelling, as well as tender joints, swollen joints, and both tender and swollen joints, showed significantly higher values of joint uptake ratio (*p* < 0.05 for all).

**Fig. 3:**
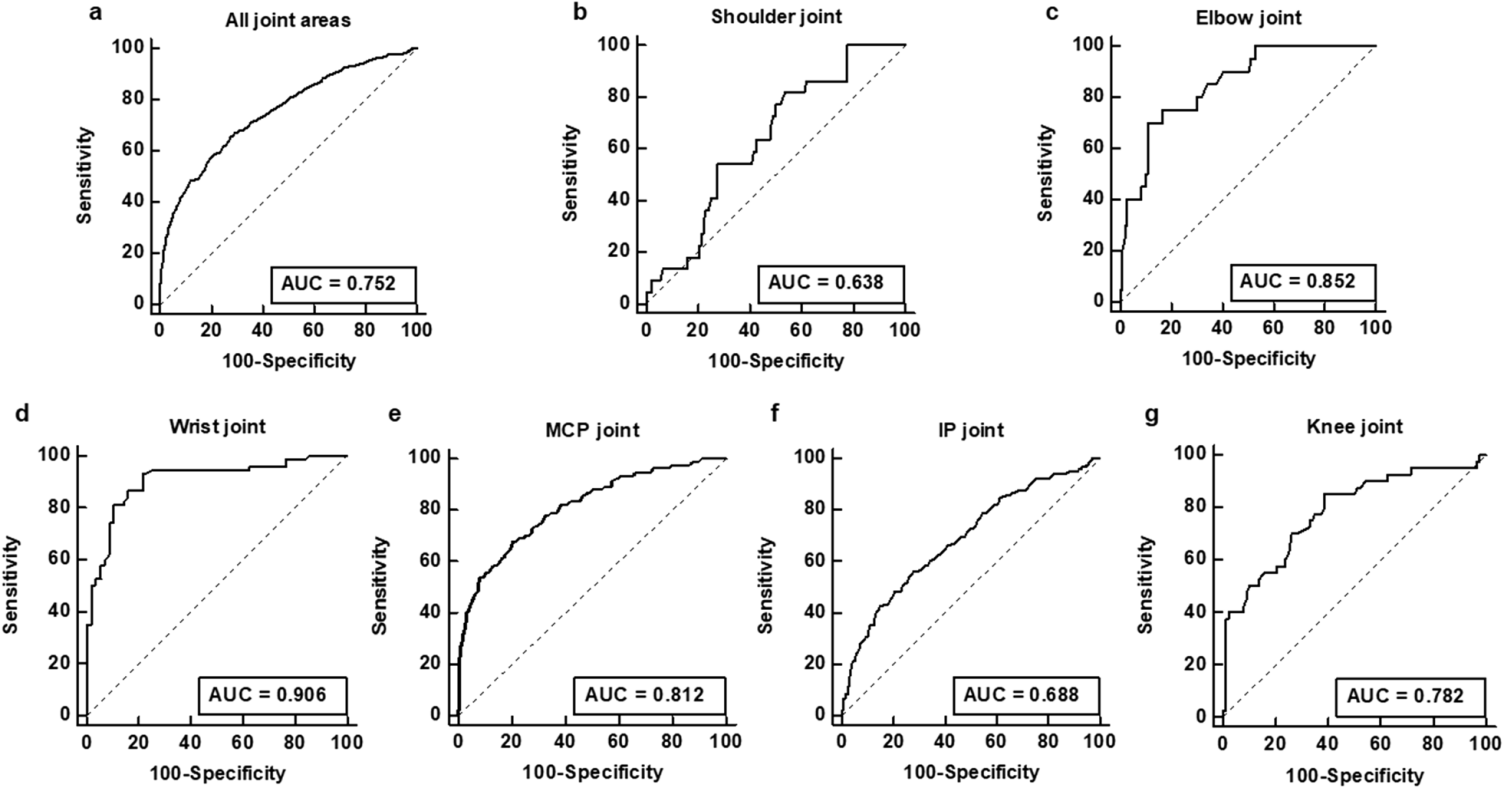
Receiver operating characteristic curves for the joint uptake ratio for identifying clinically active joints among the 1817 joints (**a**), 130 shoulder joints (**b**), 129 elbow joints (**c**), 130 wrist joints (**d**), 650 metacarpo-phalangeal (MCP) joints (**e**), 650 interphalangeal (IP) joints (**f**), and 128 knee joints (**g**)

The detection ability of joint uptake ratio for clinically active joint is shown in Table 2. For 1817 joints included, the joint uptake ratio showed a sensitivity of 78.7% (95% CI, 74.5–82.5%), specificity of 52.0% (95% CI, 49.4–54.7), positive predictive value of 32.9% (95% CI, 31.3–34.6%), and negative predictive value of 89.1% (95% CI, 87.1–90.8%) with a cut-off value of 1.07 determined by ROC curve analysis (AUC, 0.752; 95% CI, 0.724–0.778; Fig. 3a). With regard to the detection ability of the joint uptake ratio for each of six joint areas, high sensitivity and negative predictive value for detecting clinically active joints in all six joint areas (shoulder, elbow, wrist, MCP, hand IP, and knee; Fig. 3b–g), especially for the elbow (AUC, 0.852; 95% CI, 0.754–0.925), wrist (AUC, 0.906; 95% CI, 0.843–0.950), and MCP joints (AUC, 0.812; 95% CI, 0.754–0.857) were observed.

**Table 2:** The ability of joint uptake ratio in detecting clinically active joint

Joint	AUC (95% CI)	Cut-off joint uptake ratio	Sensitivity (%) (95% CI)	Specificity (%) (95% CI)	PPV (%) (95% CI)	NPV (%) (95% CI)
Shoulder (n = 130)	0.638 (0.527–0.751)	1.47	81.8 (59.7–94.8)	46.3 (36.7–56.2)	23.7 (19.3–28.8)	92.6 (83.4–96.9)
Elbow (n = 129)	0.852 (0.754–0.925)	1.31	75.0 (50.9–91.3)	83.5 (75.2–89.9)	45.5 (33.7–55.7)	94.8 (89.5–97.5)
Wrist (n = 130)	0.906 (0.843–0.950)	1.86	93.2 (84.9–97.8)	78.6 (65.6–88.4)	85.2 (77.6–90.5)	89.8 (78.9–95.4)
MCP (n = 650)	0.812 (0.754–0.857)	1.23	81.3 (71.8–88.7)	62.1 (57.9–66.1)	25.9 (23.2–28.7)	95.3 (93.0–96.9)
Hand IP (n = 650)	0.688 (0.641–0.740)	0.90	76.0 (68.9–82.2)	47.4 (42.8–52.0)	34.0 (31.4–36.8)	84.7 (80.7–88.0)
Knee (n = 128)	0.782 (0.678–0.863)	1.37	85.0 (70.2–94.3)	61.4 (50.4–71.6)	50.0 (42.7–57.3)	90.0 (80.9–95.0)
Total (n = 1817)	0.752 (0.724–0.778)	1.07	78.7 (74.5–82.5)	52.0 (49.4–54.7)	32.9 (31.3–34.6)	89.1 (87.1–90.8)

AUC, area under the receiver operating characteristic curve; CI, confidence interval; IP, interphalangeal; MCP, metacarpo-phalangeal; NPV, negative predictive value; PPV, positive predictive value

### Summed joint uptake ratio for detecting high disease activity

**Fig. 4:**
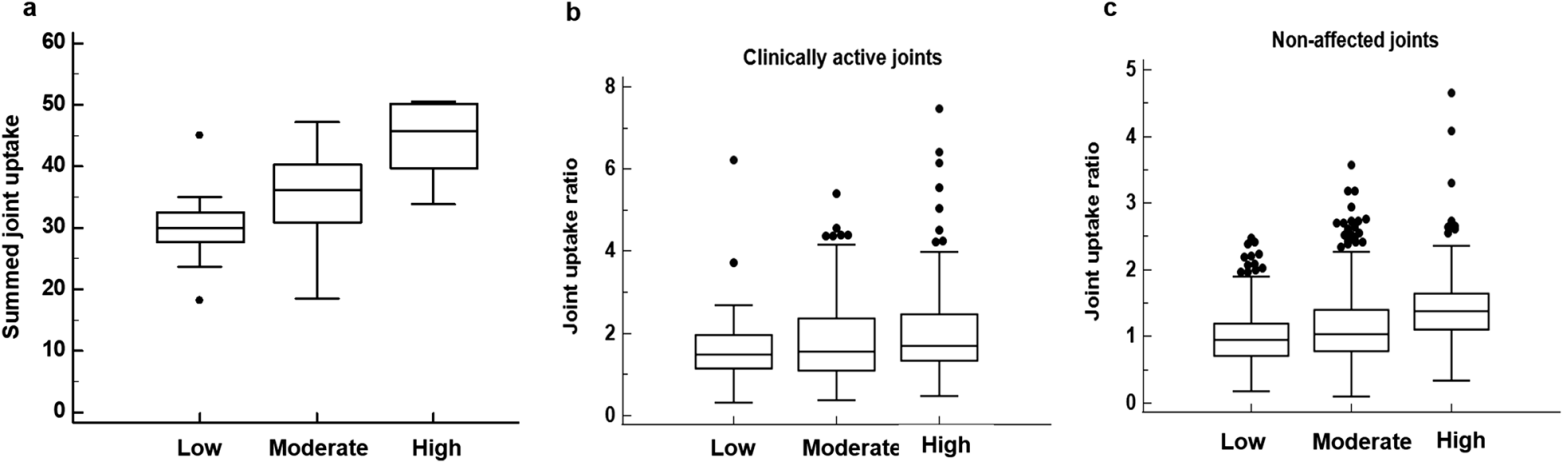
Distribution of the summed joint uptake ratio of the 28 joints (**a**), joint uptake ratio of the clinically active joints (**b**), and joint uptake ratio of the non-affected joints (**c**) according to the disease activity of rheumatoid arthritis (Low, DAS28-ESR ≤ 3.2; Moderate, 3.2 < DAS28-ESR ≤ 5.1; High, DAS28-ESR > 5.1)

Comparison of summed joint uptake of the 28 joints according to disease activity revealed that there were significant differences in summed joint uptake ratios among patients with high, moderate, and low disease activities (*p* < 0.001; Table 3; Fig. 4a). Post-hoc comparisons showed that patients with high, moderate, and low disease activities showed significant differences in summed joint uptake ratios (*p* < 0.05). Furthermore, summed joint uptake ratios of the 28 joints in patients with high, moderate, and low disease activities were significantly higher than that in comparison patient group with osteoarthritis (*p* < 0.05 for all; Table 3).

**Table 3:** Comparison of joint uptake ratio and summed joint uptake ratio of the 28 joints between patients with high, moderate, and low disease activities and patients in comparison group

Disease activity	Joint uptake ratio		Summed joint uptake ratio of 28 joints
	Clinically active joints	Non-affected joints	
High (DAS28-ESR >5.1)	1.96 ± 1.13	1.38 ± 0.54	44.8 ± 5.8
Moderate (3.2< DAS28-ESR ≤5.1)	1.82 ± 0.96	1.12 ± 0.49	35.4 ± 7.5
Low (DAS28-ESR ≤3.2)	1.65 ± 0.86	0.99 ± 0.38	30.1 ± 5.6
*P*-value	0.115*	<0.001†	<0.001*
Comparison group patients with osteoarthritis	1.94 ± 0.90	0.88 ± 0.35	27.1 ± 3.3
*P*-value	0.273^‡^	< 0.001^‡^	< 0.001^‡^

DAS28-ESR, disease activity score in 28 joints using erythrocyte sedimentation rate

^*^P-values for analysis of variance between patients with high, moderate, and low disease activities

^†^*P*-value for the Kruskal–Wallis test between patients with high, moderate, and low disease activities

^‡^*P*-value for the Kruskal–Wallis test between patients with high, moderate, and low disease activities, and patients in comparison group with osteoarthritis

We further compared joint uptake ratio of clinically active joint and non-affected joint among patients with high, moderate, and low disease activities and patients in comparison group to investigate the cause of the difference in summed joint uptake ratio according to disease activity (Table 3). With regard to clinically active joint, although joint uptake showed a tendency to increase with an increase in disease activity, there was no significant difference in joint uptake ratio according to disease activity (*p* = 0.115; Fig. 4b). Further, irrespective of disease activity, clinically active joint showed no significant differences of joint uptake ratio with affected joints in comparison group with osteoarthritis (*p* > 0.05 for all). Meanwhile, for non-affected joints, there was a significant difference in joint uptake ratio according to disease activity (*p* < 0.001; Fig. 4c), and post-hoc comparisons demonstrated that patients with high, moderate, and low disease activities showed significant differences in joint uptake ratios (*p* < 0.05). In comparison with patients in the comparison group, non-affected joints in patients with rheumatoid arthritis showed significantly higher values of joint uptake ratio than non-affected joints in patients with osteoarthritis irrespective of disease activity (*p* < 0.05 for all).

On Correlation analysis, the summed joint uptake ratio of the 28 joints showed a significantly strong positive correlation with DAS28-ESR (*p* < 0.001; correlation coefficient, 0.725; 95% CI, 0.585–0.824). Furthermore, summed joint uptake ratio also revealed moderate positive correlations with serum ESR level (*p* < 0.001; correlation coefficient, 0.462; 95% CI, 0.246–0.634) and patient global assessment (*p* < 0.001; correlation coefficient, 0.511; 95% CI, 0.305–0.671).

**Fig. 5:**
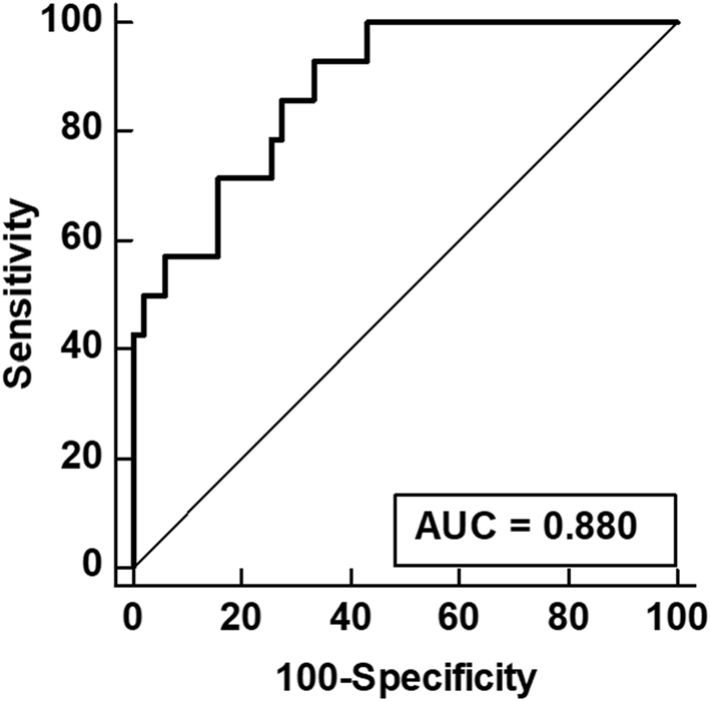
Receiver operating characteristic curves for the summed joint uptake ratio of the 28 joints for detecting high disease activity among the enrolled patients with rheumatoid arthritis

On ROC curve analysis, the summed joint uptake ratio of 28 joints showed high detection ability for high disease activity in patients with rheumatoid arthritis (AUC, 0.880; 95% CI, 0.775–0.947; Fig. 5). The optimal cut-off values of 36.3 for summed joint uptake ratio showed the sensitivity, specificity, positive predictive value, and negative predictive value of 92.9% (95% CI, 66.1–99.8%), 66.8% (95% CI, 52.1–79.2%), 43.3% (95% CI, 33.6–53.6%), and 97.1% (95% CI, 83.6–99.6%), respectively, for detecting high disease activity.

## Discussion

In the present study, we investigated the clinical value of the quantitative parameter of bone scintigraphy in the assessment of patients with rheumatoid arthritis. Bone scintigraphy is known to have high sensitivity in detecting active arthritis on the basis of hyperemia and bone erosion in an inflamed joint [6, 17]. However, because the Tc-99m diphosphonate accumulates in all joint areas to some degree, even in joints with no pain and swelling, there has to be inevitable difficulty in differentiating joints with synovitis by visual interpretation [13, 17]. In a previous study, there was a moderate inter-rater agreement in the visual analysis of joint uptake classification, with a kappa coefficient of 0.597, and only 63.4% agreement was found between two independent readers for categorizing joints with a moderate increase in uptake [10]. Meanwhile, quantitative analysis can provide an objective and reliable parameter for estimating the degree of joint uptake on bone scintigraphy [10, 15], and the joint uptake ratio used in the current study showed a substantial inter-reader agreement in our previous study, showing a concordance correlation coefficient of 0.987 [10]. The results of our study demonstrated that joint uptake ratio was significantly higher in clinically active joints than in non-affected joints, suggesting the role of joint uptake ratio in discriminating inflamed joints in patients with rheumatoid arthritis. Furthermore, the summed joint uptake ratio of the 28 joints had a significant positive correlation with DAS28-ESR and serum inflammatory marker levels, indicating that patients with high, moderate, and low disease activities showed significant differences in summed joint uptake ratios. Because patients with high disease activity would tend to have greater numbers of clinically active joints than other patients, it might be obvious that those with high disease activity had higher values of summed joint uptake ratio. However, one of the interesting findings in our study is that patients with high disease activity had significantly higher joint uptake ratios in non-affected joints than others, whereas no statistical significance was shown for the comparison of joint uptake ratio in clinically active joints according to disease activity. Considering that a significant proportion of joints without clinical arthritis in patients with rheumatoid arthritis is found to have subclinical joint inflammation [18, 19], patients with high disease activity might have a greater number of joints with subclinical inflammation, which also contributed to an increase in summed joint uptake ratio in patients with high disease activity. Furthermore, irrespective of disease activities, non-affected joints in patients with rheumatoid arthritis showed significantly higher values of joint uptake ratios than non-affected joints in the comparison group with osteoarthritis, indicating the presence of subclinical inflammation in a substantial proportion of joints in patients with rheumatoid arthritis in spite of disease activities. Until now, findings of joints without active arthritis on bone scintigraphy has received only a little attention, but the results of our study suggest that joint uptake ratios of non-affected joints might provide a clue for differential diagnosis of rheumatoid arthritis.

In the present study, the joint uptake ratio showed high sensitivity and negative predictive value for identifying clinically active joints throughout the whole joint areas, however, the detection ability seems to be different among the joint areas. The joint uptake ratio showed a high detection ability with an AUC of > 0.800 for the wrist, elbow, and MCP joint areas, while a low detection ability with an AUC of < 0.700 for shoulder and hand IP joint areas. It has been already reported that increased joint uptake on bone scintigraphy could be observed in the shoulder joint area without clinical arthritis [20], which would limit the diagnostic value. Further, gamma cameras with routine thallium-doped sodium-iodide detectors have a potential problem of image quality deterioration in imaging small joints of the hands because of a suboptimal spatial resolution and poor anatomical definition [21], which might limit the proper quantification of uptake in hand IP joint areas. In contrast to the high sensitivity and negative predictive value, the joint uptake ratio showed a low specificity and positive predictive value in almost all joint areas. It is well-known that etiological diagnosis of arthritis cannot be made in accordance with the degree of joint uptake on bone scintigraphy images and the results of our study also revealed no significant difference of joint uptake ratio of clinically active joints between patients with rheumatoid arthritis and osteoarthritis, which results in a lack of specificity [6, 10, 17]. Furthermore, as shown in the results of our study, an increase in joint uptake ratio was observed even in non-affected joints among patients with high disease activity, showing an overlapping of joint uptake ratio between clinically active joints and non-affected joints, which could further lower the specificity and differential diagnostic value of joint uptake ratio. Considering the low specificity and high negative predictive value of joint uptake ratio, the quantitative analytic method of bone scintigraphy could have a clinical role as a screening imaging test in patients with joint symptoms during initial work-up, rather than in the differential diagnosis of joint involvement in patients with arthritis [10].

Previously, only a few studies have measured the joint uptake ratio for whole-body joint areas on bone scintigraphy in patients with rheumatoid arthritis [13, 16]. Unlike the results of the present study, previous studies that conducted quantitative analysis demonstrated that there were no significant differences in joint uptake ratio according to the clinical degree of arthritis, and the sensitivity and specificity for detecting clinically active arthritis did not significantly differ between quantitative and visual analyses [13, 16]. However, these previous studies were performed almost three decades ago with only a small number of patients (< 20 patients), and all of the enrolled participants had undergone treatment for rheumatoid arthritis before bone scintigraphy [13, 16]. In contrast, we enrolled a fair number of patients upon initial work-up with no previous history of rheumatoid arthritis treatment, which could have contributed to the differences between the results of the current and previous studies. Further studies with a larger population are needed to validate the clinical value of quantitative analytical method of bone scintigraphy.

In patients with rheumatoid arthritis, the use of ultrasonography and MRI is recommended for the initial work-up to improve the certainty of diagnosis and to identify joints with clinical arthritis [7]. Ultrasonography and MRI have significant clinical values in detecting joint inflammation and monitoring treatment response [22–24]. However, visual analysis is mainly used for the interpretation of ultrasonography and MRI, and consequently, discrepancies between readers are still a major concern [23–25]. Meanwhile, using our in-house software, bone scintigraphy can automatically provide objective and quantitative parameters of whole-body joint areas within a few minutes. Using the cut-off values of joint uptake ratios provided in the present study, bone scintigraphy analyzed with our technology could provide sites of clinically active joint in the whole-body area, which could bring focus of physician’s attention to the overlooked joint areas and could indicate the anatomical sites where further assessment using other imaging examinations such as MRI and ultrasonography is needed. Also, considering the results of comparing joint uptake ratios of non-affected joints between patients with rheumatoid arthritis and osteoarthritis, our quantitative analytic method might help with differential diagnosis of rheumatoid arthritis. In addition, the strong positive correlation between the summed joint uptake ratio and DAS28-ESR suggests that quantitative parameters of bone scintigraphy might be used as imaging biomarkers for estimating disease activity in future studies with rheumatoid arthritis. Notwithstanding, there is a possibility of difficulty in applying our analytic method to patients with long-lasting active arthritis, distorted joints, and anatomical deformity [10], which could limit the general use of quantitative analysis. Recently, with the development in medical technology, the semiconductor cadmium zinc telluride detectors and ultra-fast whole-body single-photon emission computed tomography have been introduced in bone scintigraphy imaging [21, 26, 27]. These new technologies might pave the way to overcome this limitation and enhance the use of quantitative analysis on bone scintigraphy.

There are several limitations to the present study that needed to be addressed. First, the enrolled patients were retrospectively selected from a single center; therefore, further validation of our results is needed. Second, age-range of the participants in our study was broad, and because of the retrospective nature of the study, we could not classify the patients according to the clinical degree of joint inflammation or symptom duration, which might have affected the findings of bone scintigraphy [10, 25]. Lastly, only the ability of quantitative analysis of bone scintigraphy for identifying clinically active joints and detecting high disease activity were assessed in the study. To establish the clinical role of this quantitative analytic method, further prospective studies that compare its clinical value with other imaging modalities and evaluate the impact on management planning are needed.

## Conclusions

The joint uptake ratio, the quantitative parameter measured using bone scintigraphy, showed high sensitivity and negative predictive value for identifying clinically active joints in patients with rheumatoid arthritis, and showed a high detection ability especially in the wrist, elbow, and MCP joints. Furthermore, the summed joint uptake ratio of the 28 joints showed strong positive correlation with DAS28-ESR and had high sensitivity and negative predictive value for detecting patients with high disease activity. Quantitative parameters of bone scintigraphy could provide valuable information regarding joint involvement of active arthritis and disease activity in patients with rheumatoid arthritis; however, further studies are needed to validate the results of the current study.

## Data Availability

The datasets used and/or analysed during the current study are available from the corresponding author on reasonable request.

## Abbreviations

ACPA: Anti-citrullinated peptide antibody
AUC: Area under the receiver operating characteristic curve
CI: Confidence interval
DAS28: Disease activity score of 28 joints
DAS28-ESR: Disease activity score of 28 joints using erythrocyte sedimentation rate
ESR: Erythrocyte sedimentation rate
IP: Interphalangeal
MCP: Metacarpo-phalangeal
MRI: Magnetic resonance imaging
NPV: Negative predictive value
ROC: Receiver operating characteristic
